# Integrative taxonomy on the fast track - towards more sustainability in biodiversity research

**DOI:** 10.1186/1742-9994-10-15

**Published:** 2013-03-27

**Authors:** Alexander Riedel, Katayo Sagata, Yayuk R Suhardjono, Rene Tänzler, Michael Balke

**Affiliations:** 1 Museum of Natural History Karlsruhe (SMNK), Erbprinzenstr, Karlsruhe, 13, D-76133, Germany; 2 Papua New Guinea Institute for Biological Research (PNG-IBR), Goroka, Papua New Guinea; 3 Zoological Museum, Cibinong Science Center - LIPI, Jl. Raya, Jakarta- Bogor, Indonesia; 4 Zoological State Collection, Münchhausenstr, Munich, 21, D-81247, Germany; 5 GeoBioCenter, Ludwig-Maximilians-University, Munich, Germany

**Keywords:** Taxonomic impediment, Integrative taxonomy, DNA barcoding, Taxonomic description

## Abstract

**Background:**

A so called “taxonomic impediment” has been recognized as a major obstacle to biodiversity research for the past two decades. Numerous remedies were then proposed. However, neither significant progress in terms of formal species descriptions, nor a minimum standard for descriptions have been achieved so far. Here, we analyze the problems of traditional taxonomy which often produces keys and descriptions of limited practical value. We suggest that phylogenetics and phenetics had a subtle and so far unnoticed effect on taxonomy leading to inflated species descriptions.

**Discussion:**

The term “turbo-taxonomy” was recently coined for an approach combining *cox1* sequences, concise morphological descriptions by an expert taxonomist, and high-resolution digital imaging to streamline the formal description of larger numbers of new species. We propose a further development of this approach which, together with open access web-publication and automated pushing of content from journal into a wiki, may create the most efficient and sustainable way to conduct taxonomy in the future. On demand, highly concise descriptions can be gradually updated or modified in the fully versioned wiki-framework we use. This means that the visibility of additional data is not compromised, while the original species description -the first version- remains preserved in the wiki, and of course in the journal version. A DNA sequence database with an identification engine replaces an identification key, helps to avoid synonyms and has the potential to detect grossly incorrect generic placements. We demonstrate the functionality of a species-description pipeline by naming 101 new species of hyperdiverse New Guinea *Trigonopterus* weevils in the open-access journal ZooKeys.

**Summary:**

Fast track taxonomy will not only increase speed, but also sustainability of global species inventories. It will be of great practical value to all the other disciplines that depend on a usable taxonomy and will change our perception of global biodiversity. While this approach is certainly not suitable for all taxa alike, it is the tool that will help to tackle many hyperdiverse groups and pave the road for more sustainable comparative studies, e.g. in community ecology, phylogeography and large scale biogeographic studies.

## Background

Species hypotheses are the basic currency of comparative biology, yet a major portion of global biodiversity remains unnamed and thus in the dark [1]. Remedies for overcoming the taxonomic impediment include the increased development of human resources and new technological approaches [2,3]. Tools from a taxonomists’ wish list ranging from powerful imaging technologies and DNA sequencing to fast and open internet access are now widely available. Nevertheless, significant progress in terms of formal species descriptions has not been achieved to date. Instead, a decline in taxonomic productivity per author has occurred since World War II [4,5]. The reasons for this decline are complex, but often the desire to include as many characters as possible in the original description of a new species increases their average length and decreases their number. Nevertheless, issues of quality control could not be addressed sufficiently in traditional taxonomy because morphological descriptions are difficult to standardize. This leads to the problem of synonymy which requires continued efforts to be fixed [6]. Furthermore, lack of standards also means that extremely uninformative descriptions are still being published, which further complicates matters - and does not help to improve the image of the whole discipline.

### The practice of taxonomic description

We suggest that the advent of phylogenetic systematics [7] and phenetics [8] had a profound but little-noticed effect on the preparation standards of species descriptions. Since more and more taxonomic revisions incorporated phylogenetic analyses or were at least prepared in parallel with the latter, it was attempted to maximize the number of informative characters. Thus, even characters of little value for species diagnosis were included in the descriptions. Another consequence was that species descriptions within a study were sought to be standardized, best illustrated by the program Delta [9]. Negative character states (i.e. the absence of a character) were often explicitly stated. Thus, the average length of species descriptions increased and their number per author decreased in the past 50 years [4,5]. Often enough, all this time-consuming procedure did not enhance the usability of descriptions for the purpose of diagnosis, but rather inflated them. After all, standardization among different authors was never achieved not to mention the failure to introduce an urgently needed minimum standard.

### Taxonomic impediment or impediment to taxonomy?

The “taxonomic impediment” is known as the situation in which biological studies suffer from shortcomings of the taxonomic basis, i.e. the difficulty in safely identifying many species [10]. We propose that the vast number of undescribed species on Earth [11] may not be the biggest problem in this context. A name and a safe diagnosis for a new species can be provided rapidly and with limited resources. The bigger problem is usually the legacy of earlier taxonomic work, i.e. the interpretation of existing names. Many descriptions are inadequate and to clarify matters, the type specimens have to be examined. The revision of a minor taxonomic group may require extensive travel to museums around the world, without a guarantee that the critical characters are actually found on the types. For example, if a diagnosis based on male characters is state of the art, there is little help if some of the species were described based on unique female specimens. One of the oldest principles of nomenclature, i.e. the Principle of Priority apparently promotes “taxonomic mihilism” (from Latin *mihi* – belonging to me) [12]: the taxon’s earliest description ensures the name’s use, no matter how low the diagnostic value of the associated description is. Authors with a strong mihi-itch have described new taxa based on inadequate material or data, just to secure authorship of the species; the ensuing problems for identification are left to be sorted out by the community. In orphaned taxa without a sufficient number of experts, taxonomic data of heterogeneous quality become a heavy burden rather than a tool for identification. We suggest that these self-inflicted and system-inherent problems are the main reason for the taxonomic impediment, possibly closely followed by a lack of determination of many biodiversity research projects to include a sufficient budget for taxonomic work.

It appears as a sad irony that a part of the taxonomic community [13,14] turns a blind eye on these problems while blaming any constructive criticism from end-users [2,15] as the true impediment to taxonomy. Below we propose that turbo-taxonomy can effectively combine the strengths of both traditional, morphology-based taxonomy and DNA based approaches. We emphasize that a good quality of work always depends on the standards of the persons involved and that the use of DNA sequences is no insurance against over-splitting or other mistakes. But, the combination of morphology and DNA taxonomy will allow to assess and solve such problems more easily than before.

### The approach

#### Examples of turbo-taxonomy

**Figure 1:**
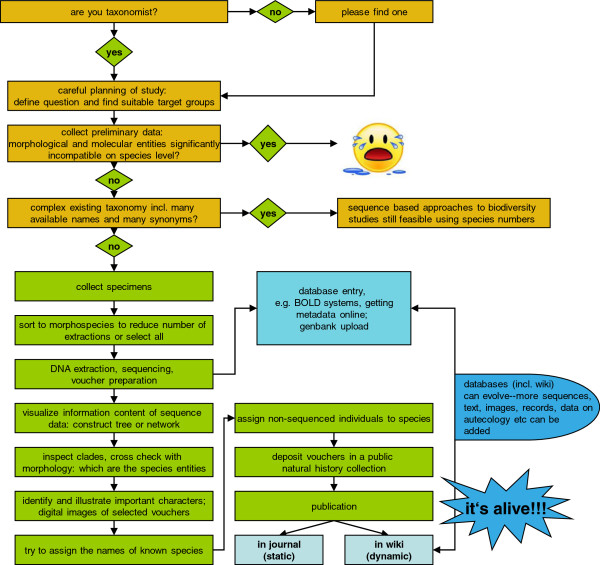
Flow chart of the turbo-taxonomy approach, from project design to publication.

The term “turbo-taxonomy” was coined for an approach combining DNA barcoding with short taxonomic descriptions of morphological characters for hyperdiverse parasitic wasps [16]. We extend this approach by abstaining from laborious, but not necessarily helpful identification keys, and rather adding automated journal-wiki upload (pushing) of data, to reveal and formally describe 101 species of hyperdiverse *Trigonopterus* weevils. Thus, we combine traditional expert taxonomy with DNA sequencing, subrobotic digital imaging (where a machine takes images of different specimen layers and stacks them automatically) and automated content pushing from a journal into a wiki to show explicitly how to sustainably provide species with the attributes that makes them most visible: names anchored in a framework more rapidly produced than currently the case [17]. Concatenated, versioned species pages using the wiki engine offer a continuous opportunity for subsequent enhancement and community participation (Figure 1).

We established the genus *Trigonopterus* as our first target for comparative biodiversity studies because it is highly diverse within a region of great biological interest, both genetically and in terms of species. We collected >6,000 specimens of *Trigonopterus* from across New Guinea and sequenced 1,000 of them, assigned to 279 entities of putative species status [18,19]. We showed that mitochondrial and nuclear DNA entities were indeed fully congruent or compatible with morphologically delineated groups and argue that such widespread congruence within a taxon is the most important prerequisite for an accelerated framework (Figure 1). The judgment of species status was mainly based on examination of male genital characters. Morphologically delineated species with high *cox1* divergence were examined a second time, and nuclear DNA markers sequenced to discover potentially diagnostic nDNA characteristics that suggest the existence of “cryptic” species or reveal overlooked species. The final hypotheses incorporate evidence from both morphology and molecules. After a preliminary screening of known *Trigonopterus* types, we here avoided the risk of creating synonyms by excluding the few species that could potentially bear a valid name. Species represented only by females were preliminarily excluded, as additional field work may later discover males which we prefer as holotypes. All 279 species are clearly delineated as can be seen in the maximum likelihood tree based on *cox1* sequences of 1,002 specimens of *Trigonopterus* [link to http://www.plosone.org/article/fetchSingleRepresentation.action?uri = info: doi/10.1371/journal.pone.0028832.s001] [19]. We formalize our findings by describing the first 101 species new to science [20], introducing a condensed format fully embracing technological advances and in accordance with the International Code of Zoological Nomenclature [21,22]. As an example, we include this description from the ZooKeys paper.

### *Trigonopterus phoenix* Riedel

**Figure 2:**
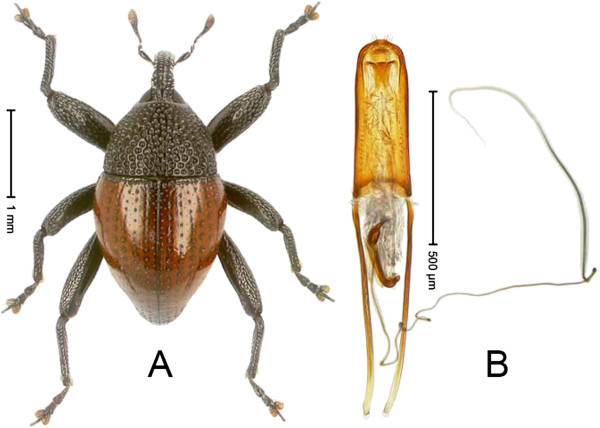
***Trigonopterus phoenix *Riedel (A) Habitus (B) Aedeagus.**

Holotype, male (Figure 2A, http://species-id.net/wiki/Trigonopterus_phoenix. Length 2.63 mm. Beetle black; antennae, tarsi and elytra ferruginous. Body subovate; with weak constriction between pronotum and elytron; in profile evenly convex. Rostrum in basal half with distinct median ridge and pair of submedian ridges, furrows with sparse rows of yellowish scales; apically weakly punctate, sparsely setose. Pronotum coarsely punctate-reticulate. Elytra with distinct striae of small punctures; intervals with row of minute punctures; laterally behind humeri with ridge bordered by 4 deep punctures of stria 9. Femora edentate. Mesofemur and metafemur dorsally squamose with silvery scales. Metafemur with weakly denticulate dorsoposterior edge; subapically with stridulatory patch. Metatibia apically with uncus and minute premucro. Abdominal ventrite 5 coarsely punctate, in apical half with round depression fringed with dense erect scales. Aedeagus (Figure 2B) apically weakly pointed, sparsely setose; transfer-apparatus spiniform; ductus ejaculatorius with bulbus. Intraspecific variation. Length 2.53–2.63 mm. Female rostrum in apical half slender, dorsally subglabrous, with sublateral furrows. Female abdominal ventrite 5 densely punctate, with suberect scales, with median ridge.

Material examined. Holotype (SMNK): ARC1153 (EMBL # HE615781), PAPUA NEW GUINEA, Simbu Prov., Karimui Dist., Haia, Supa, S06° 39.815' E145° 03.169' to S06° 39.609' E145° 03.012', 1240–1450 m, 30-IX-2009. Paratype (NAIC): PAPUA NEW GUINEA, Simbu Prov., ARC1132 (EMBL # HE615761), S06° 40.078' E145° 03.207' to S06° 39.609' E145° 03.012', 1220–1450 m, 02-X-2009.

Notes. This species was coded as “Trigonopterus sp. 207” by Tänzler et al. (2012).

Etymology: From the ancient Greek Φοίνιξ, “the reborn”.

**Figure 3:**
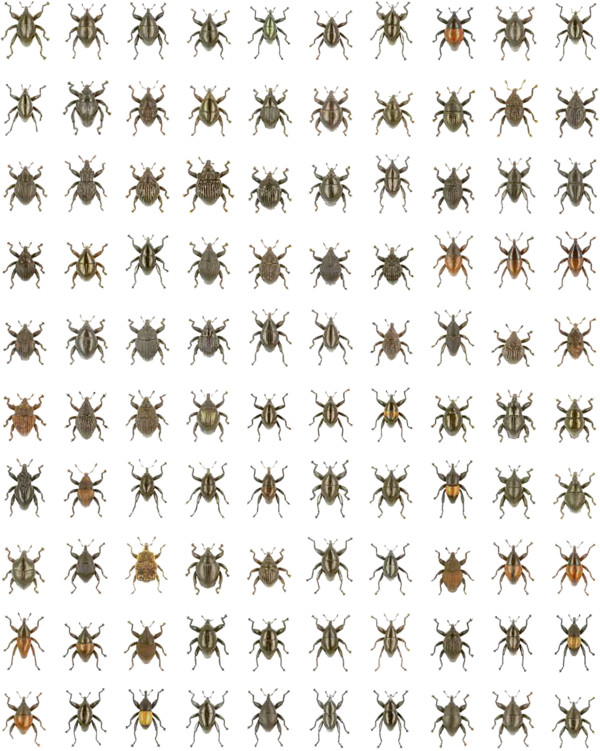
**Compilation of 100 new *Trigonopterus *species.**

This species and 100 additional ones (Figure 3) were described simultaneously in the open-access journal ZooKeys [20]. Holotypes were designated exclusively from sequenced specimens. Photographs of habitus and genitalia were prepared after DNA extraction from holotypes. Thus, potential confusion by type series of mixed species is excluded by providing all relevant data from the holotype.

## Discussion

A combination of digital imaging and molecular techniques allows the reduction of formal species descriptions to brief but highly accurate diagnoses. Although none of these tools is novel in itself, the progressive element is their combination and streamlining to produce a large number of usable species descriptions.

### DNA barcoding

The potential of using a *standard* DNA marker for species identification, also known as “DNA barcoding” or “DNA taxonomy”, was recognized almost ten years ago [3,23]. Despite fierce initial and some continued criticism it proved to be a powerful tool. In many animal taxa, the “barcoding” sequence (usually *cox1*) will pinpoint the correct species without additional information [24,25]. In others it may not delineate species unambiguously, but even then it is usually possible to determine a group of e.g. 5–10 species [26]. A non-expert would hardly achieve this level of accuracy within reasonable time using traditional keys on most invertebrate taxa, let alone nematodes, moss mites or rove beetles. After all, *in combination* with a few morphological characters the species can be safely identified in most cases. Furthermore, sequence data can be easily databased, searched, analyzed and accessed anytime from anywhere. The situation with type specimens is quite different: often they are not accessible, or it is very time-consuming to send them around the globe. In many cases they give the only clue what species an insufficient description is referring to, or if the species is placed in the correct genus at all. Such issues are common and could be solved much faster using “DNA barcodes”. We strongly believe that the ICZN should make the publication of genetic data obligatory following the example of the “Bacteriological Code” [27] which stipulates taxon-specific requirements for a meaningful and valid description of new extant species. On the downside such a decision would mean that material stored in collections could no longer be used for most taxonomic purposes as soon as its DNA is degraded. However, in many cases it is still possible to extract and sequence DNA from historic specimens [28], and if not, it may be an option to collect fresh material. Surely, this would bring taxonomists more often to the field than is currently the case. On the upside, the new descriptions published would be of greater value and would cause less headache to the community (see above “Taxonomic Impediment or Impediment to Taxonomy?”). Realistically, taking a look at the Code’s pace of change, we anticipate that such a decision is still decades away. Until that day, the contest between descriptions containing DNA barcodes and the ones without may give an answer of what data is really needed.

### Online databases and wikis

**Figure 4:**
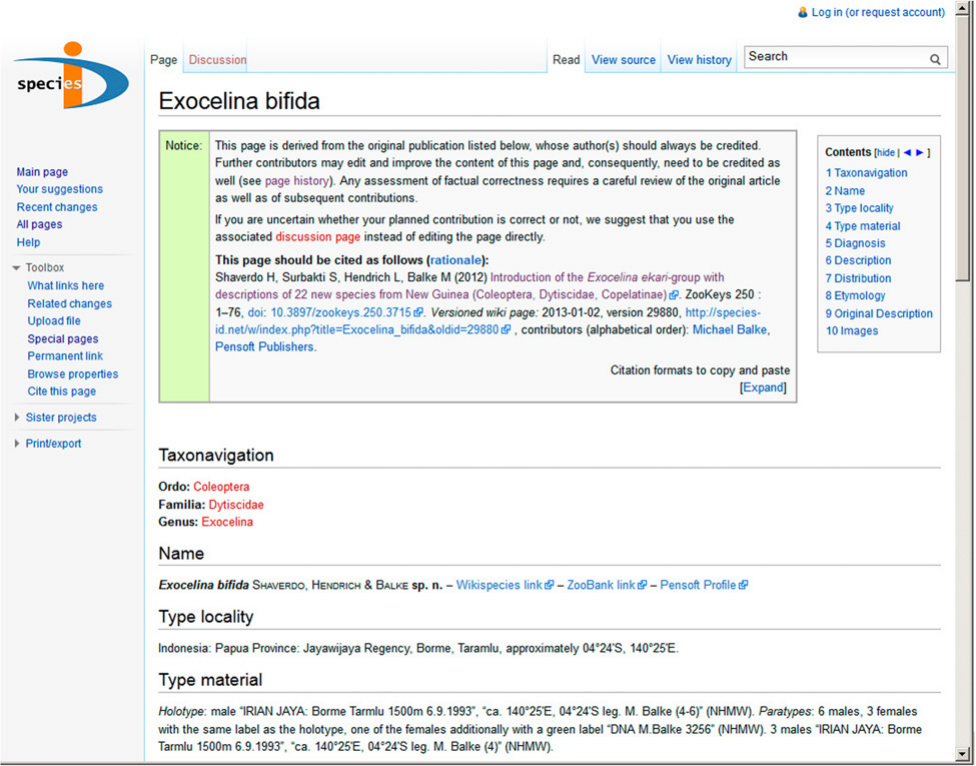
Screenshot of the upper part of a Species-ID wiki species page, showing the notice box which contains author credits and full citation of the page.

**Figure 5:**
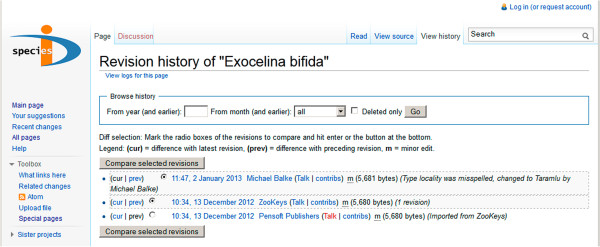
Screenshot of the revision history feature of a Species-ID wiki species page; here, a first minor edit was made on a newly uploaded page.

Online wiki databases such as the Species-ID portal [link to http://species-id.net/wiki/] [29] are not recognized as means of publication by the International Code of Zoological Nomenclature [21,22], so their significance requires some explanation here. The open-access journal “ZooKeys” has pioneered a publication format that makes a new name available with a traditional paper publication [30], but simultaneously creates a versioned wiki with the same content [31]. There is a notice field on top of each page (Figure 4) which provides credits and a reference to the original source, and the wiki framework allows monitoring the editing history (Figure 5) [32]. ZooKeys pushes all taxon treatments at genus and species rank to Species-ID. Transferred data include highly resolved illustrations which then can be used to zoom into details. This wiki can be updated later anytime with additional data, be it an elaborate 3D-model or a “quantum contribution” [33] such as a simple collecting record. We currently update our first ca. 30 pages with additional images and DNA sequence data from a phylogenetic study (in the diving beetle genus *Exocelina*). At the time the species becomes formally named there is no urgency to provide the description with all possible data. It should contain a reasonable basis, so that its diagnosis is guaranteed. But most users will later rather consult the online working description, gradually being supplemented with additional data. Thus, the formal species description is like a healthy newborn which is expected to grow into an adult with the help of its environment. In the case of *Trigonopterus*, characters such as the functional morphology of thanatosis or the morphology of the metendosternite, surely of great interest but of little diagnostic value, can be added at a later stage without compromising their visibility - meaning they are attached to the original reference, versioned so that the sequence of text changes remain visible. In general, we believe that the wiki format is the best platform for species pages [34], and purpose-built pages such a Species-ID can easily be linked and connected to wiki species to increase visibility. With billions of page requests per annum, it also appears safe to assume that the wiki environment will not easily disappear.

As apparent from the latest changes of the ICZN regarding online descriptions [22] the official registry of zoological nomenclature ZooBank [link to http://zoobank.org/] [35] may at some stage take a central role in a unitary taxonomy [2]. If taxonomic descriptions could be published within ZooBank as envisioned by Minelli [36] the restrictions of this database-system would also speak for an initial minimalistic description including diagnostic sequence data. The majority of barcoding sequences currently contained in GenBank are not identified to species [37] and environmental sequencing will not improve this situation; also, many of the GenBank entries in general may indeed represent misidentifications [38]. A database with sequences derived mainly from holotypes would necessarily have a much higher reliability. Unless mistakes in the sequencing process or the handling of sequence data are discovered [39], these sequences would not change, just as the original nomenclatural data. Thus, these data would surely fit well into the concept of ZooBank. At a time when the idea of open-source is spreading and researchers begin to see dissemination of their works as their obligation and not as a source of income, the biggest problem towards a unitary taxonomy may have disappeared already. If a suitable infrastructure was provided by ZooBank, a critical mass of researchers would start uploading images, diagnosis-texts and sequences to obtain immediate publication and permanent storage on an Official Database of Zoological Nomenclature. The ICZN should team up together with major natural history museums around the world, provide the necessary cyber-infrastructure and make additional relevant changes to the Code. The BOLD system [link to http://www.boldsystems.org] [40] could serve as a source of inspiration, because data upload is easy, and each individual can have its own voucher page with images that show what the voucher looks like, maps where it comes from, collecting data, sequences, trace files and most importantly information where the voucher physically IS (e.g. Voucher of *Batrachedra praeangusta* link to http://http:/www.boldsystems.org/index.php/Public_RecordView?processid=LBCH3416-10.

### An integrative fast track approach

It is hard to quantify the amount of time needed for an average description, and to compare the traditional approach with ours. Actual manuscript preparation (i.e. descriptions and photographs, names, and listing of specimens) of the 101 species took about one year which is equivalent to the time needed for a traditional revision of 10–15 species [41,42]. We estimate that our fast track approach leads to an increase of about 5 to 10 times compared to traditional, comprehensive descriptions. This does not include laboratory work associated with DNA extraction, sequencing and sequence analysis. However, such work does not need to be performed by the taxonomist whose time is usually the limiting factor. The processing of about 1000 specimens took about six weeks of laboratory work and subsequent sequence data analysis. Naturally, the precise amount of time saved by the fast track approach depends on the taxonomic group and on the personal style of the taxonomist, but we believe that an acceleration rate of 2–20 times can be achieved for many hyperdiverse taxa.

In the following we discuss seven factors that contribute to a higher effectiveness of turbo taxonomy compared to traditional taxonomic work:

1) Easier sorting process of species by the availability of an underlying molecular phylogeny. Sorting a long number of small specimens belonging to many similar species is like playing a memory matching game of a thousand similar cards with a microscope. If the scaffold of molecular data is at hand, comparison of the morphology can be limited to the specimens of close genetic similarity. Pre-publication “synonyms” leading to the preparation of duplicate data can be avoided in the process. This concerns especially specimens from different localities as the sorting of morphospecies is most effective within a given locality sample.

2) Renouncement on the preparation of a traditional identification key. For a large number of similar species it is time-consuming to prepare keys based on morphological characters. One example of “turbo-taxonomy” [16] contains such a key, but we believe that this is contradictory to the idea of DNA barcoding or acceleration of taxonomy in hyperdiverse taxa. Usually, it is possible to divide a larger number of species into clear-cut groups. However, closely related species are often distinguished by complex genital characters difficult to describe in words and even more difficult to translate into a dichotomous key. The same applies for subtle differences, e.g. of the surface sculpture. Unless a key is provided with numerous illustrations there remains a high degree of ambiguity, often a serious problem even for an expert of a specific group. Furthermore, the presence of many unknown species to be added to a key later considerably reduces its practical value.

3) Reduction of the description to essential diagnostic characters. Relatively unimportant characters that are often just added to make descriptions formally comparable are omitted.

4) Reduction of the description of “intraspecific variation”. Series of length measurements quoting averages and standard deviation are extremely time-consuming and in most cases of no value for the purpose of diagnosis. Usually, it will be sufficient to measure a few specimens representing the extremes known at the time of description.

5) Reduction of the number of illustrations. Highly resolved images retain a lot of detailed information if they are published online, instead of printed relatively small in size. Arrangements of overviews and details as required by printed plates become superfluous. Different aspects of one species would often be desirable, but the added value of such multiple images decreases compared to descriptions of different species. We found that in our case two images per species have the highest information content/time ratio.

6) Comparative diagnoses are redundant: The selection which species are compared side-by-side is highly subjective. Characters differentiating from the species with relatively close genetic similarity should be covered by the morphological description.

7) Tracing and interpreting historic type specimens can be extremely time-consuming. In our case, some of this work was done already, and some could be avoided by our selection of species to be described. To maintain a universal taxonomy, it will be necessary to invest more time and money to provide existing names with DNA barcodes. Once this is done, future taxonomists would need to spend just a fraction of the time and travel funds needed now on tracing and examining type specimens.

This brings us to the main target of our approach - which taxa are most suitable? Turbo-taxonomy will work best if either a high proportion of existing species are present in the sequence database, or, if only a small proportion have been described so far (Figure 1). The latter case we expect in many tropical arthropods. In groups with a long history of study and a wealth (respectively load) of existing taxonomic names the situation is different: the time needed to tag existing species with DNA sequences may outweigh the time saved in the process of describing new species. Nevertheless, a long number of described species is not necessarily an indication that a barcoding approach would not be effective. The genus *Conotrachelus* Dejean 1835 with a staggering number of ca. 1,200 described species still shows a high proportion of undescribed species on a local scale [43]. In such cases it is more a question of how large a drafted project may become with given resources. The expert taxonomist will know best how many new species of a given taxon to expect and what difficulties the tagging of existing species may pose. Based on our own experience we are confident that a significant number of taxa highly suitable for “turbo-taxonomy” will be found.

## Conclusion

In 1758, the big bang of zoological taxonomy [44] came with a key to all animal life then known and by providing 2 to 3-line descriptions. We firmly believe that technology provides researchers with suitable tools for completing Linnaeus’ work much more rapidly and with more sustainable, better results than those currently obtained. DNA sequences provide the “key element”, while web-based illustrations and short diagnoses should be sufficient to define the name and face of a species.

We question the prevailing taxonomic practice of preparing long, time-consuming descriptions of often-irrelevant morphological characters and making great efforts to prepare static identification keys that are often useless to non-experts and that become obsolete after the discovery of additional species. A dynamic (e.g.) *cox1* sequence database with an identification engine efficiently replaces traditional keys and helps to avoid both synonymy and grossly incorrect generic placements (i.e. might stimulate the researcher to re-assess morphological characters), thus contributing to a more sustainable taxonomy.

Our approach shows that traditional taxonomic expertise and new technology are perfectly compatible, creating a taxonomy more transparent and sustainable than ever before. It would at last allow us to tackle groups with an overwhelming diversity of similar species that taxonomists still tend to shy away. This would surely change our perception of global biodiversity and would be of great practical value to all the other disciplines that depend on a usable taxonomy.

## Competing interests

The authors declare that they have no competing interests.

## Authors’ contributions

MB and AR designed the study. AR, YS and KS performed fieldwork. RT performed the molecular work and analyzed sequences; AR defined the morphospecies and prepared descriptions. All authors participated in manuscript preparation; all have read and approved the final manuscript.

## Authors’ information

MB, AR and YS are museum curators and have each published many “conventional” species descriptions in the form of revisions over the past two decades. Later in their career they started using molecular data, having learned their lessons.
